# Clinical characteristics and correlation analysis of IVIG resistance in children with kawasaki disease complicated with hip synovitis: case-control study

**DOI:** 10.3389/fped.2023.1193722

**Published:** 2023-06-27

**Authors:** Jianjie Wang, Xing Rong, Huixian Qiu, Yue'e He, Maoping Chu, Zhenquan Wang

**Affiliations:** Children's Heart Center, The Second Affiliated Hospital and Yuying Children’s Hospital, Institute of Cardiovascular Development and Translational Medicine, Wenzhou Medical University, Zhejiang, China

**Keywords:** kawasaki, Synovitis, ROC, Logistic, IVIG resistance

## Abstract

**Objective:**

To investigate the clinical characteristics and risk factors of Kawasaki disease (KD) complicated with hip synovitis.

**Methods:**

Children with KD admitted from January 1, 2011, to December 31, 2020, in the KD database of Yuying Children's Hospital Affiliated with Wenzhou Medical University were retrospectively included. We selected KD children with hip synovitis as the case group and KD children without hip synovitis as the control group to analyze the possible risk factors of hip synovitis in KD children.

**Results:**

Among 2,871 KD children admitted to our center in recent years, 28 had hip synovitis. In this study 140 KD children were enrolled, including 28 KD children with hip synovitis and 112 children with general KD (within one month of admission). The onset age of KD patients with hip synovitis was 30.92 (23.23–49.99) months, and there were 17 cases of bilateral hip involvement. The course of synovitis (limited movement, joint pain, lameness, unwillingness to stand, etc.) ranged from 1 to 19 days, with an average of (8.8 ± 4.6) days. We treated all KD children with IVIG (Intravenous immunoglobulin) plus aspirin, among which five patients in the case group developed coronary artery damage, six acquired IVIG resistance, and synovial inflammation disappeared within two weeks. Age, weight, length of stay, and incidence of IVIG resistance significantly differed between the two groups (*P* = 0.001, 0.005, <0.001, and 0.035, respectively). Logistic regression analysis showed that KD combined with hip synovitis was an independent risk factor for developing propyl pellet resistance, with an OR value of 4.625 (95% CI: 1.095, 19.526).

**Conclusion:**

KD combined with hip synovitis mainly involves bilateral hip joints, and joint pain and limited movement are the main clinical features. The symptoms are mild and self-limiting. KD combined with hip synovitis is a risk factor for IVIG resistance. Hip synovitis is a good predictor of IVIG resistance.

## Introduction

The etiology of Kawasaki disease (KD) is unknown, and it affects multiple systems throughout the body. These effects include coronary artery damage, pancreatitis, aseptic meningitis, synovitis, etc (1). The risk of cardiovascular involvement after KD is a significant cause of morbidity and mortality. Many studies have reported cardiovascular events in KD, but other complications of KD, such as synovitis, have not been registered. Synovitis is clinically characterized by swelling and fluid collection of the joints with typical inflammatory symptoms such as redness, pain, or fever (2). Temporary hip synovitis is a benign, self-limiting disease. Children with hip synovitis often have movement pain, limited walking, and synovial effusion but no noticeable swelling of the joint appearance (3, 4). The treatment of KD has been relatively mature (5, 6). A study in Japan showed that about 10%–20% of KD children did not respond to IVIG treatment (7), higher than that of children sensitive to IVIG treatment (8). Many KD children with hip synovitis in our center developed resistance to IVIG, but the specific pathophysiological mechanism remains unclear. This study analyzed whether KD combined with hip synovitis was a risk factor for IVIG resistance by comparing KD children without hip synovitis and KD children with hip synovitis, which might provide a theoretical foundation for early detection, timely intervention, and improved recovery.

## Subjects

**Figure 1:**
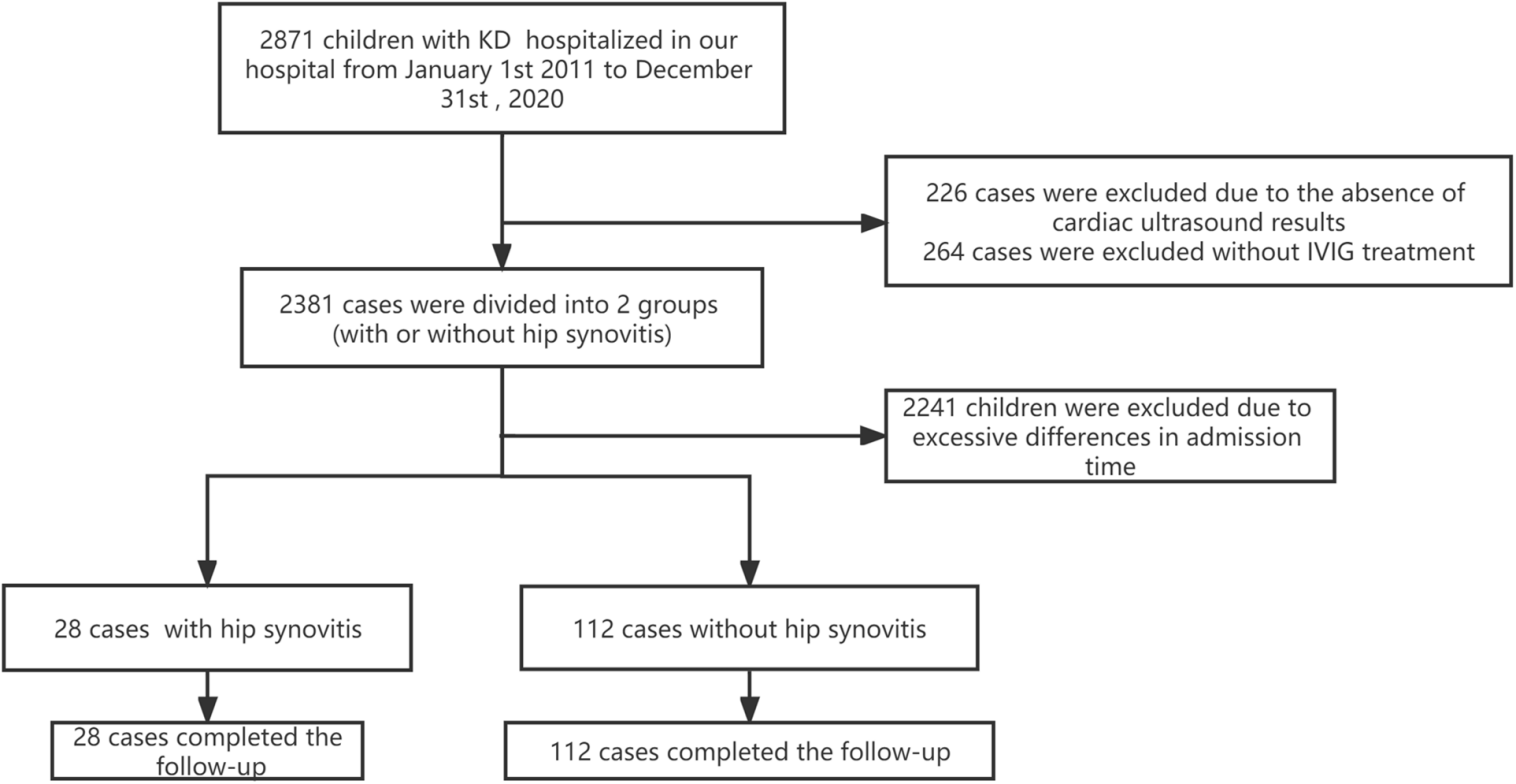
Patients flow chart. Flow chart showing the demographic and clinical information of all study participants. We enrolled 2871 children in our KD databases from 1 January 2011 to 30 December 2020. Four hundred ninety cases were excluded due to the lack of IVIG treatment or cardiac ultrasound results. The remaining patients were divided into two groups according to the presence or absence of hip synovitis, of which 28 patients had hip synovitis (case group). We excluded 2,241 KD children due to the significant difference between the admission time and the case group. Finally, 28 KD children complicated with hip synovitis and 112 common KD were enrolled in this study.

**Figure 2:**
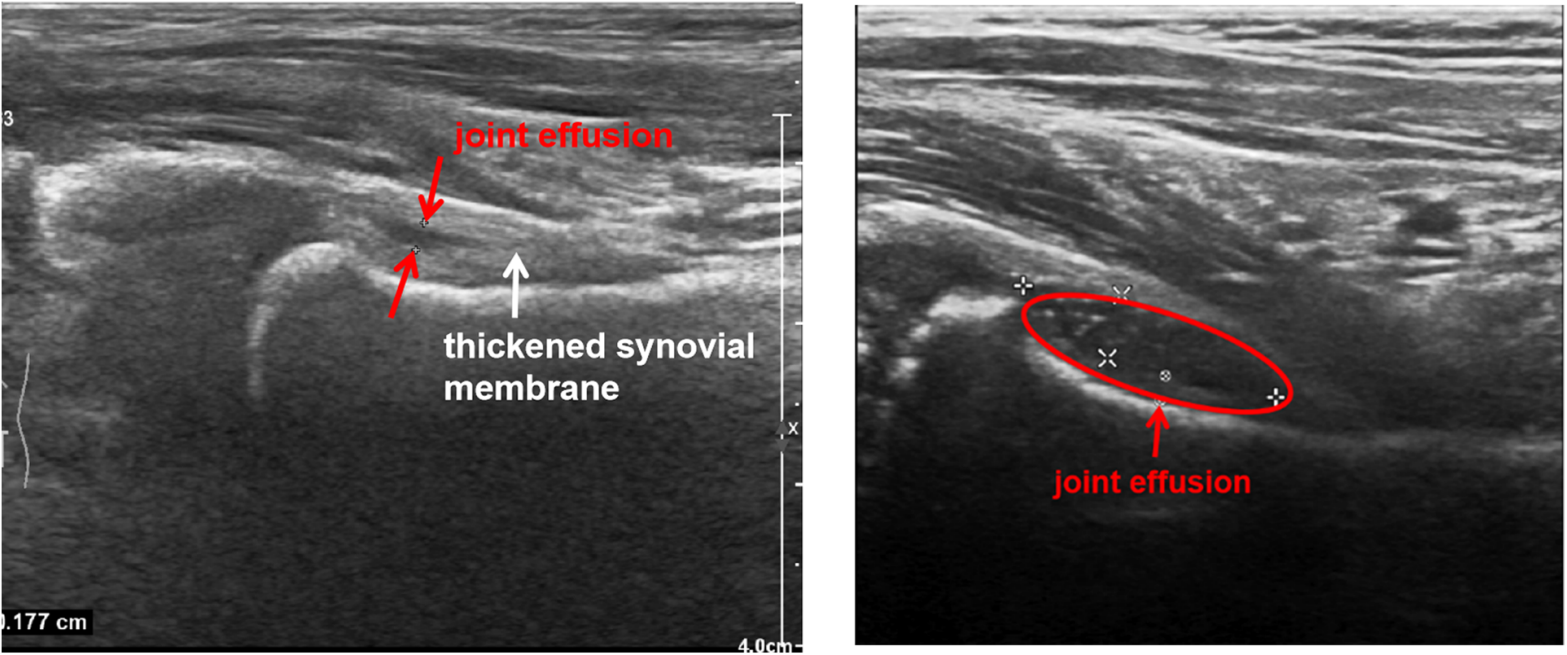
Ultrasound results of kawasaki disease in children with hip synovitis.

We reviewed 2,871 children with KD who were hospitalized in our hospital from January 1st, 2011, to December 31st, 2020, which included 28 KD children who were diagnosed with hip synovitis. At the same time, we selected 112 children hospitalized at the same time as the case group (with a difference of 1 month before and after hospitalization) as shown in Figure 1. All KD children followed the Japanese diagnostic criteria (9), and the diagnosis of hip synovitis was mainly based on hip ultrasound (4), as shown in Figure 2. Then we observed the two groups' general demographic characteristics, laboratory indicators, length of hospital stay, and duration of IVIG use. We adjusted corresponding confounding factors to determine whether KD combined with hip synovitis was a risk factor for IVIG resistance.

## Statistics

The distribution of age, body weight, and length of hospital stay in the two groups showed skewed distribution. The rank sum test and gender adoption rate index were adopted to analyze the gender difference by Chi-square test. Among laboratory indicators, Hemoglobin (Hb), Absolute Neutrophil Count (ANC), and Albumin (Alb) samples were in line with normal distribution, and we adopted a single-sample *T*-test. The indexes of Platelet (PLT), C-reactive protein (CRP), Erythrocyte Sedimentation Rate (ESR), Alanine aminotransferase (ALT) and Brain natriuretic peptide (BNP) showed skewed distribution, and we used the rank sum test. As for the clinical features of the children, such as changes in limb swelling and lymph node enlargement, and the incidence of CAL and IVIG resistance between the two groups, the rate was taken as the indicator, and the Chi-square test was used to compare the difference in the rate. In addition, ROC curve analysis was performed to obtain the critical value of independent risk indicators to analyze children with IVIG resistance, and the optimal CUT value was brought based on the ROC curve. Variables were converted into dichotomy variables for hierarchical analysis, and each layer's corresponding P and OR values were calculated.

### Types of Kawasaki disease

Complete KD is defined as having at least four clinical manifestations except for persistent fever for five days (9). Incomplete KD is defined as having two clinical manifestations except for persistent fever and coronary artery damage or three clinical manifestations except for persistent fever (9).

### Efficacy of intravenous gamma globulin

IVIG resistance was defined as persistent fever ≥38.0°C after 48 hours of IVIG treatment, otherwise IVIG sensitivity (10).

## Results

A total of 140 children diagnosed with KD were included in this study, 28 children were clinically diagnosed with hip synovitis through hip MRI, hip B-ultrasound, or clinical presentation of lower limb walking difficulties. The median age of the KD with synovitis was 30.92 months [interquartile range (IQR): 23.23–49.99 months], 57.1% was male, 75% was complete KD, 78.6% was sensitive to IVIG, 17.9% presented coronary artery lesions. There were significant differences in median weight and hosday between the two groups (*P* = 0.005, *P* < 0.001). Regarding clinical manifestations, the risk of lymph node enlargement, limb joint swelling, and lip changes did not increase. Meanwhile, no difference was found in other laboratory indicators such as Hb, ALB, ALT, and BNP. There were also significant differences in IVIG resistance between the two groups, among which the rate of IVIG resistance of KD with synovitis was 21.4%, higher than that in Normal KD children (*P* = 0.035). Table 1 shows the specific clinical characteristics.

**Table 1:** Clinical characteristics of the 140 children with kawasaki disease.

	Synovitis (*N* = 28)	Normal (*N* = 112)	*P* value
Age (months)	30.92 (23.23, 49.99)	22.64 (13.57, 36.74)	0.001
Male (*n*, %)	16 (57.1%)	56 (50.0%)	0.499
Weight (kg)	13.5 (12.0, 18.0)	12.0 (10.0, 15.0)	0.005
Swelling of extremities	21 (75%)	94 (83.9%)	0.27
Rash	22 (78.6%)	100 (89.3%)	0.202
Lymphadenopathy	18 (64.3%)	59 (52.7%)	0.269
Oral lesions	28 (100%)	103 (92%)	0.204
IKD	7 (25%)	19 (17.1%)	0.339
Hosday	10.5 (8.25, 13.71)	8.0 (7.0, 10.0)	<0.001
CAL (%)	5 (17.9%)	25 (22.3%)	0.607
IVIG Resistance (%)	6 (21.4%)	7 (6.3%)	0.035
PLT (×10^12^/L)	361.50 (269.00, 434.50)	358.00 (276.25, 416.25)	0.872
ANC (×10^9^/L)	12.62 ± 5.65	10.65 ± 4.98	0.071
CRP (mg/L)	89.00 (55.85, 159.63)	60.46 (37.09, 101.87)	0.016
Hb (g/L)	113.50 ± 13.29	113.69 ± 10.39	0.936
ESR (mm/h)	38.50 (33.75, 56.00)	36.00 (28.00, 46.00)	0.074
ALB (g/L)	39.41 ± 5.65	39.92 ± 4.63	0.619
ALT (IU/L)	45.00 (13.00, 159.00)	38.00 (18.00, 126.25)	0.49
NT-proBNP (×10^6^/L)	458.5 (187.75, 3312.50)	898.5 (355.50, 2182.50)	0.294

**Figure 3:**
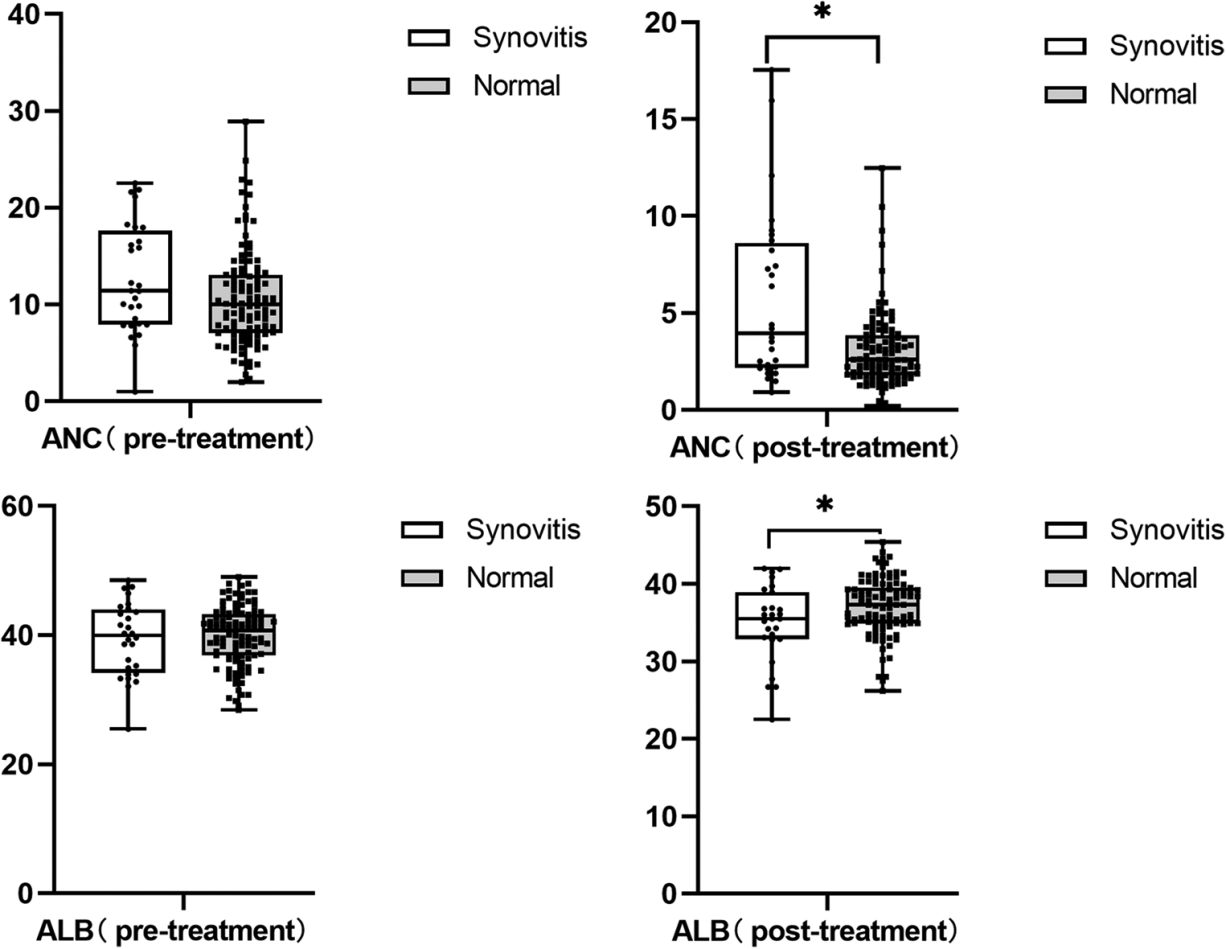
Albumin and neutrophil levels after iVIG treatment.

We also found that children with hip synovitis had higher neutrophil levels (*P* = 0.006) and lower albumin levels (*P* = 0.016) after IVIG treatment, as shown in Figure 3. To further evaluate the relationship between hip synovitis and IVIG resistance in children with KD, we performed a multivariate analysis using IVIG resistance as the outcome variable. Indicators including whether there was hip synovitis, gender, age of the child, incomplete Kawasaki, albumin level, ALT level, platelet level, standard treatment, delayed diagnosis and treatment were included. After excluding corresponding confounding factors, it was found that KD children complicated with hip synovitis were more likely to cause no response to IVIG treatment, as shown in Table 2. In addition, to ensure the reliability of the results, the ROC curve was used to select the optimal CUT value for predicting IVIG resistance, and the consequences of stratified analysis also supported the above conclusions, as shown in Table 3.

**Table 2:** Risk of IVIG resistance in children with KD combined with hip synovitis.

Exposure	Non-adjusted	Adjusted		
		Model 1	Model 2	Model 3
Synovitis of hip	4.091 (1.253, 13.357)	3.947 (1.175, 13.260)	4.650 (1.271, 17.018)	4.625 (1.095, 19.526)
	0.020	0.026	0.020	0.037

Value is expressed as OR value (95% CI) *P* value.

Model 1 adjusted for age (months, ≤60 months, >60 months) and gender (male, female).

Model 2 adjusted for: model 1 + C-reactive protein level (≤70 mg/L, >70 mg/L), platelet count (≤450 × 109/L, >450 × 109/L), alanine aminotransferase level (≤45 U/L, >45 U/L).

Model 3 adjusted for: model 2+ Kawasaki disease type (complete, incomplete)+ treatment regimen(standard, non-standard), and time of IVIG treatment(delayed, non-delayed).

**Table 3:** Stratified analysis of IVIG resistance in children with KD.

Stratification factor	*N*	ALL patients (*N* = 140)	
		OR (95% CI)	*P*-value
**C-reactive protein level**			
≤121.38 mg/L	116	4.769 (1.186, 19.184)	0.028
>121.38 mg/L	24	2.667 (0.208, 4.197)	0.451
**Albumin level**			
≤40 g/L	68	2.833 (0.425, 8.877)	0.282
>40 g/L	72	5.4 (1.156, 25, 225)	0.032
**Platelet count**			
≤450*10^9^/L	24	2.125 (0.152, 29.659)	0.575
≤450*10^9^/L	116	4.889 (1.281, 18.657)	0.02
**Absolute neutrophil count level**			
≤15.41*10^9^/L	114	4.667 (1.160, 18.778)	0.03
>15.41*10^9^/L	26	3.111 (0.245, 39.540)	0.382

## Discussion

As a complication of various diseases, hip synovitis is not uncommon. Lohmander, L.S. reported the pathogenesis of synovitis as early as 1988 (11), and it was also reported in patients with hemophilia and varicella (12, 13).In recent years, hip synovitis has also been found in pregnant women complicated with COVID-19 (14), and as a complication of Kawasaki disease (15, 16). Previous research showed the incidence rate for transient synovitis was 76.2 per 100,000 person-years (17). In our study, the rate was 97.5 per 100,000 person-years. (Among 2,871 children with Kawasaki disease admitted to our center in 10 years, 28 had hip synovitis).

Kawasaki disease with hip synovitis could express with joint pain, lameness, and even walking impairment. In our study, 14 presented with joint pain, 12 with limited mobility, 4 with claudication, and 2 with positive hip quadrangular signs. Juvenile idiopathic arthritis (JIA) has been reported with hip synovitis (18, 19). Due to the lack of corresponding characteristic biomarkers, the differential diagnosis of JIA and KD is more challenging when the condition occurs. IL-1b is a mediator of synovial inflammation and has a suggestive effect on injury or stress. After stimulation, serum IL-1b levels are elevated in JIA (20). Data from animal studies in KD suggest that IL-1b is involved in developing inflammation, intravenous immunoglobulin administration, and cardiac outcome events (21, 22). Takahara T et al. found that elevated serum IL-18 levels helped differentiate JIA from KD (23).

The characteristics of transient hip synovitis are interesting. MarjoleinKru et al. found that the peak age of onset of transient hip synovitis appears between 4 to 10 years, and boys are twice more than girls to develop transient hip synovitis (TSH) (17). Our results showed that children with KD combined with hip synovitis were older, heavier, and had a more extended mean hospital stay. We suspect that age may explain this clinical feature, with older children having more movement and a larger hip cavity, which is more likely to cause fluid accumulation in the joint. In addition, current studies have shown that KD is mainly caused by host immune dysfunction, leading to vascular endothelial injury. Besides the coronary artery, the lesions can also involve other blood vessels in the whole body, resulting in blood supply disorders of corresponding organs. The occurrence of hip synovitis may also be related to iliac artery disease.

Synovitis of the hip can be used to assess the level of inflammation in the body, which helps predict non-response to IVIG treatment (24, 25). IVIG resistance is often regarded as a feature of severe cases. It has been confirmed to be related to the occurrence and development of CAL in many studies (26, 27). Peng Hu et al. found that plasma cytokines such as IL-6 and TNF-α were involved in KD inflammation during the acute phase, and the count levels of inflammatory factors in non-responders were significantly higher than those in IVIG responders (24), which was consistent with our conclusion. Our study found that KD children with hip synovitis showed a higher IVIG resistance rate. ANC, CRP, and ESR levels were higher than those of normal KD children, and the levels of ALB were lower after treatment. In addition, we included clinical features, laboratory indicators, and other factors to construct a Logistic model of hip synovitis and IVIG resistance. After excluding confounding factors, it was found that children with hip synovitis had a higher risk of IVIG resistance. IVIG resistance was used as a predictor to construct a ROC curve further to test this factor's influence in different populations. Optimal CUT values were selected for CRP, ALB, PLT, and ANC levels and then stratified. The results of the stratified analysis also supported our conclusions. Therefore, hip synovitis is a good predictor of inflammation levels and the development of IVIG resistance.

We found that all KD children with hip synovitis had a benign, self-limiting process that disappeared almost 1–2 weeks after discharge. Drugs can be used to release the pain; in our study, ibuprofen was used in 9 children with KD combined with hip synovitis, and one child with severe joint pain was treated with Diclofenac diethylamine cream and methylprednisolone pulse therapy (2 mg/kg/day) and discharged with methylprednisolone tablets (1 mg/kg/day). His arthralgia was relieved two weeks after discharge. Takuma Ito reported a case of Kawasaki disease-associated arthritis with synovial involvement. In addition to high-dose intravenous IVIG and oral aspirin, they added oral cyclosporine A on day 10 of the illness for arthritis, which was relieved on day 14 of fever (28). Pulse hormone therapy has also been used to treat Kawasaki's disease-associated synovitis (16).

The study has some limitations. Firstly, this study was a single-center study with relatively few cases. Secondly, many young children were not diagnosed due to atypical symptoms and mild joint involvement.

## Conclusions

In our study, KD combined with hip synovitis was a risk factor for IVIG resistance. Hip synovitis can be a good predictor of IVIG resistance. Therefore, clinicians should be alert to hip synovitis and improve hip ultrasound examination as soon as possible when children with KD are admitted to the hospital in the acute phase, such as walking difficulties and other manifestations. Thus, IVIG-resistant children can be identified as early as possible to reduce the occurrence of CAL.

## Data Availability

The original contributions presented in the study are included in the article/Supplementary Material, further inquiries can be directed to the corresponding author.
